# Effective use of endoscopic ultrasound for diagnosing and differentiating a gastroduodenal artery aneurysm

**DOI:** 10.1055/a-2489-8203

**Published:** 2024-12-10

**Authors:** Koichi Soga, Takeshi Fujiwara, Fuki Hayakawa, Mayumi Yamaguchi, Ikuhiro Kobori, Masaya Tamano

**Affiliations:** 1 26263 Department of Gastroenterology, Dokkyo Medical University Saitama Medical Center, Koshigaya, Japan

Aneurysms of the visceral arteries represent a rare but clinically significant vascular condition, accounting for approximately 5% of all intra-abdominal aneurysms 1 . Gastroduodenal artery aneurysms are particularly uncommon, accounting for 1.5–2.0% of all visceral artery aneurysms. Clinically, gastroduodenal artery aneurysms can present with abdominal pain and hematemesis and carry a high risk of rupture 2 . Symptoms of a gastroduodenal artery aneurysm may vary from incidental findings on a computed tomography (CT) scan to life-threatening hemorrhage due to rupture, necessitating prompt diagnosis and intervention 3 .

An 87-year-old Japanese man presented with acute renal dysfunction. His medical history was notable for hypertension and significant atherosclerosis, contributing to his renal impairment. A screening non-contrast abdominal computed tomography (CT) scan revealed a suspicious 18-mm mass adjacent to the pancreatic head. The mass was hyperdense with well-defined borders, warranting further evaluation ( Fig. 1 ). Given the patientʼs renal function, a contrast-enhanced CT was considered high-risk; thus, we opted for endoscopic ultrasound (EUS), which revealed a well-circumscribed 18-mm anechoic lesion within the pancreatic head. The lesion demonstrated continuity with tubular structures on both the cranial and caudal sides, suggestive of a vascular origin. Doppler EUS imaging confirmed an arterial waveform, leading to a diagnosis of a gastroduodenal artery aneurysm ( Fig. 2 , Video 1 ).

**Fig. 1:**
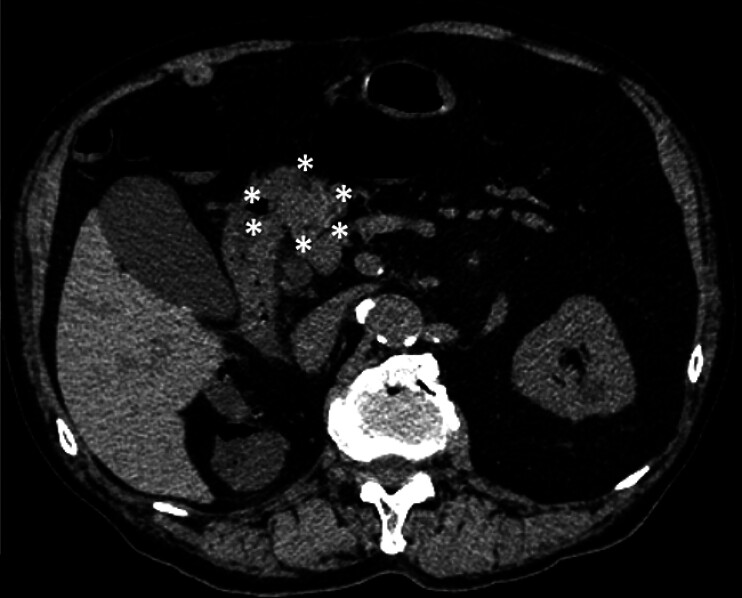
A screening non-contrast abdominal computed tomography (CT) scan revealed a suspicious 18-mm mass adjacent to the pancreatic head. The mass was hyperdense with well-defined borders, prompting further evaluation (asterisks).

**Fig. 2:**
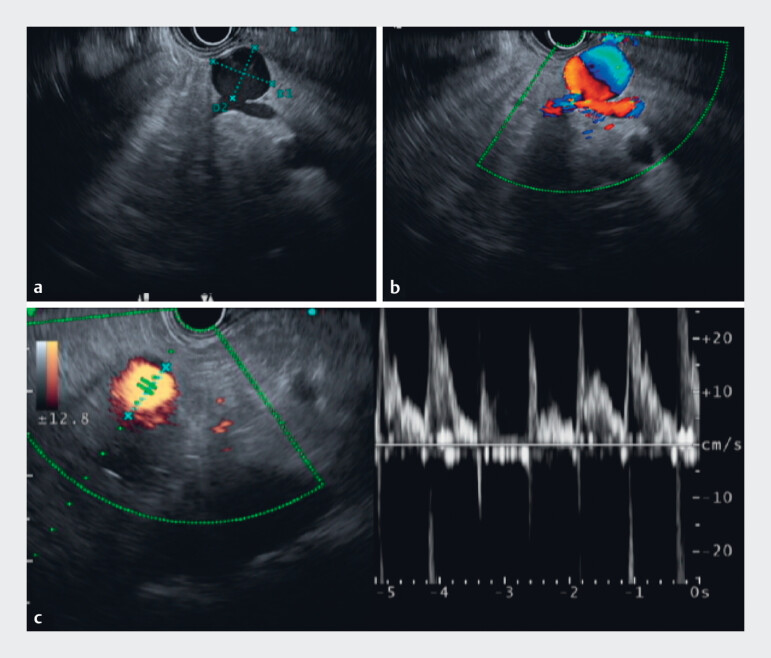
An endoscopic ultrasound (EUS) image utilizing a convex-type ultrasound for this patient. The EUS revealed a well-circumscribed, 18-mm anechoic lesion within the pancreatic head. **a** The lesion demonstrated continuity with tubular structures on both the cranial and caudal sides, suggestive of a vascular origin. **b,c** Color Doppler and power Doppler imaging confirmed blood flow within the lesion, and pulse Doppler analysis identified an arterial waveform, leading to a diagnosis of a gastroduodenal artery aneurysm.

An effective diagnostic modality of endoscopic ultrasound for the detection of gastroduodenal artery aneurysm, with differentiation from neoplastic lesions.Video 1

Non-contrast abdominal CTs may reveal only a soft tissue mass in the aneurysm bed, which can be misinterpreted as adenopathy or a neoplasm of the pancreas or duodenum. While abdominal Doppler ultrasound can identify larger aneurysms, it often lacks sufficient detail regarding the parent artery’s anatomy. Factors such as the patient's body habitus, calcified vessel walls, and limited spatial resolution can further compromise accuracy 1 .

EUS offers the necessary resolution and real-time imaging capabilities to accurately characterize lesions from closer proximity, confirming their vascular origin and leading to a correct diagnosis. This technique facilitates appropriate management planning and helps prevent potentially catastrophic outcomes associated with aneurysm rupture.

Endoscopy_UCTN_Code_CCL_1AF_2AZ

## Notes

Endoscopy E-Videos https://eref.thieme.de/e-videos
*E-Videos* is an open access online section of the journal *Endoscopy* , reporting on interesting cases and new techniques in gastroenterological endoscopy. All papers include a high-quality video and are published with a Creative Commons CC-BY license. Endoscopy E-Videos qualify for HINARI discounts and waivers and eligibility is automatically checked during the submission process. We grant 100% waivers to articles whose corresponding authors are based in Group A countries and 50% waivers to those who are based in Group B countries as classified by Research4Life (see: https://www.research4life.org/access/eligibility/ ). This section has its own submission website at https://mc.manuscriptcentral.com/e-videos .
